# Removal of volatile organic compounds using amphiphilic cyclodextrin-coated polypropylene

**DOI:** 10.3762/bjoc.10.290

**Published:** 2014-11-24

**Authors:** Ludmilla Lumholdt, Sophie Fourmentin, Thorbjørn T Nielsen, Kim L Larsen

**Affiliations:** 1 Section of Chemistry, Department of Biotechnology, Chemistry and Environmental Engineering, Aalborg University, Sohngaardsholmsvej 57, DK-9000 Aalborg, Denmark; 2 University Lille Nord de France, F-59000 Lille, France; 3 ULCO, UCEIV, F-59140 Dunkerque, France

**Keywords:** amphiphilic cyclodextrins, polypropylene, static headspace chromatography, volatile organic compounds, water purification

## Abstract

Polypropylene nonwovens were functionalised using a self-assembled, amphiphilic cyclodextrin coating and the potential for water purification by removal of pollutants was studied. As benzene is one of the problematic compounds in the Water Framework Directive, six volatile organic compounds (benzene and five benzene-based substances) were chosen as model compounds. The compounds were tested as a mixture in order to provide a more realistic situation since the wastewater will be a complex mixture containing multiple pollutants. The volatile organic compounds are known to form stable inclusion complexes with cyclodextrins. Six different amphiphilic cyclodextrin derivatives were synthesised in order to elucidate whether or not the uptake abilities of the coating depend on the structure of the derivative. Headspace gas chromatography was used for quantification of the uptake exploiting the volatile nature of benzene and its derivatives. The capacity was shown to increase beyond the expected stoichiometries of guest–host complexes with ratios of up to 16:1.

## Introduction

In October 2000, a comprehensive directive was passed by The European Parliament and Council (EU) called the Water Framework Directive, 2000/60/EC. In this directive, the member states committed themselves to prevent and reduce water pollution in order to improve the aquatic ecosystem [1]. Later in 2008, the EU elaborated on the Water Framework Directive defining several problematic compounds, including various pesticides, endocrine disruptors, and hydrocarbons such as benzene and benzene derivatives, as compounds requiring immediate and future focus. The challenge with most of these problematic compounds lies in the fact that they cannot be removed using traditional methods, for example, mechanical filtration, due to their extremely small dimensions and extremely low concentration in the wastewater. However, even low concentrations are suspected to be hazardous to the aquatic environment and/or human health [2] thus the maximum concentration limit in wastewater was set in the ppb range [1]. In the literature, various alternative wastewater treatment methods, such as treatment with active carbon or ozone, have been investigated but none could provide a full removal of the tested compounds [3]. Biological treatment of wastewater shows a high efficiency towards the removal of hydrocarbons, however, some bacteria cannot tolerate the elevated concentrations of the chemicals, resulting in a low removal efficiency [4–6]. Consequently, there is a great need to discover alternative methods to purify both industrial and private wastewater effectively in order to enable the EU member states to achieve the goals of the Water Framework Directive.

Cyclodextrins (CDs) are cyclic, relatively small molecules consisting of units of glucose connected through α-1,4-glycosidic bonds giving them a cone-like structure with a hydrophilic exterior and a relatively hydrophobic cavity [7]. The cavity is known to form inclusion complexes with several different molecules, including many of the previously mentioned, unwanted substances [8–10], which implies a potential use for CDs in water purification. The CDs can be added to water as CD polymers and filtered afterwards [11–13], however, it would be much more cost-effective to immobilise the CDs on a surface that comes into contact with the wastewater. There are several studies in the literature on the subject of immobilising CDs on surfaces with the purpose of uptake and/or release of various compounds, including applications for water purification; a selection of these are shown in Table 1 [14–20]. The methods used in these studies generally suffer from high production and running costs due to the need of advanced equipment and/or relatively low uptake capacity or low cavity coverage. Choi et al. [20] produced hollow polysulfone fibres containing CDs that were used to remove di-(2-ethylhexyl)phthalate (one of the endocrine disruptors mentioned in the Water Framework Directive). However, the fibres are exclusively produced for this purpose which might require the wastewater plant to change their existing setup and/or to buy specific equipment, all of which result in increased production costs.

**Table 1:** Examples from the literature of the use of immobilised CDs for water purification.

Technique	Support^a^	Production speed	Coating efficiency	Cavity coverage

Electron beam^b^	PP	2–26 hours depending on coating amount + drying	100–160 µmol/g	CD:guest ratio up to 1:2.3^c^
GMA mediated redox^d^	PA	2–16 hours depending on coating amount + drying	Weight gain of 1–14% depending on reaction time	Approx. ^2^/_3_ of the cavities were deemed accessible
Gamma irradiation^e^	PP + PE	2–3 days	0.5–3.1 µmol/cm^2^	CD:guest mol ratio between 1:0.6 and 1:3
Plasma^f^	PP	>72 hours	Not measured^g^	1:0.83
Layer-by-layer dip^h^	PETP	10 hours pretreatment + 2 hours per layer	Total weight gain of up to 47%	Not measured
Phase inversion/ self-assembly^i^	PS	48 hours + drying	Weight gain of up to 15%	Not measured

^a^PP: polypropylene; PE: polyethylene; PA: polyamide; PS: polystyrene; PETP: polyethylenetetraphthalate; ^b^ [14]; ^c^ [15]; ^d^ [16]; ^e^ [17); ^f^ [18]; ^g^uptake capacity, however, was measured and found to be between 3–6 µmol guest compounds per mg of treated polypropylene; ^h^ [19]; ^i^ [20].

A method for simple and fast immobilisation of CDs onto various surfaces, including polyethylene, polyvinyl chloride and polyurethane, was previously reported [21]. This method relies on the self-assembly properties of amphiphilic CDs (ACDs), which are CDs with some of the hydroxy groups substituted by alkyl chains. This can be performed in situ by, for example, simply dipping the material in question into an ethanol/water solution of a suitable ACD derivative. One of the great advantages of this technique is that it is possible to coat different materials using the same simple technique without the requirement of specialised equipment. Several possibilities for fine-tuning the coating are possible by varying the ethanol/water ratio, changing the concentration of the ACDs in the coat solution, and/or the choice of derivative [21]. It has also been shown that the coat technology can function close to industry production speeds using a kiss-roll technique [22].

In this study, it is shown how this coat technique can be used to functionalise a polypropylene (PP) filter to create a filtration membrane with a large capacity for uptake of pollutants using six different ACD derivatives. As benzene is one of the priority substances in the Water Framework Directive, benzene and five other benzene-based volatile organic compounds (VOCs) (see Table 2) were chosen as model compounds. Their uptake by the ACD-functionalised PP was characterised using static headspace gas chromatography (SH-GC). These VOCs have been shown in the literature to effectively form stable inclusion complexes with β-CD [8,23]. The VOCs were used as a mixture in order to simulate a more realistic situation since the wastewater will be a complex mixture consisting of multiple pollutants.

**Table 2:** Overview of the volatile organic compounds studied including some physical characteristics.

	**1**	**2**	**3**	**4**	**5**	**6**
	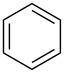	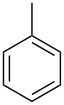	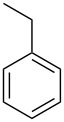	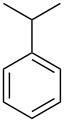	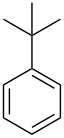	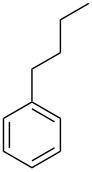

*M*_w_ (Da)^a^	78.11	92.14	106.17	120.19	134.22	134.22
Log *P*^b^	2.1	2.7	3.1	3.6	4.1	4.3

^a^ [24]; ^b^ [25].

## Results and Discussion

The six ACD derivatives were characterised with respect to the average degree of alkyl chain substitutions (DS) and location of these substitutions, as described elsewhere [21]. The structures, DS, average molecular weight, and label definitions are given in Table 3.

**Table 3:** Overview of the six amphiphilic CD derivatives used. Subs.: Number of substituents per CD molecule. DS: Degree of substitution. *M*_w_: Molecular weight.

	**7**	**8**	**9**	**10a**	**10b**	**10c**
	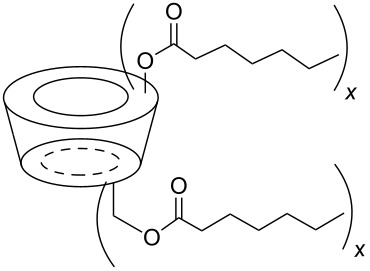	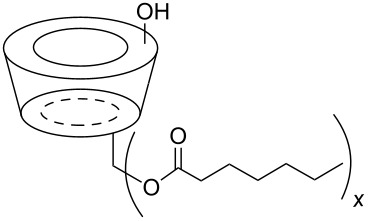	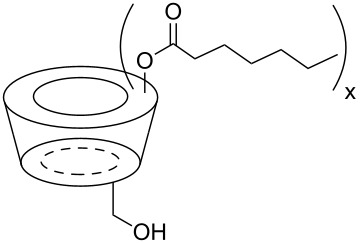	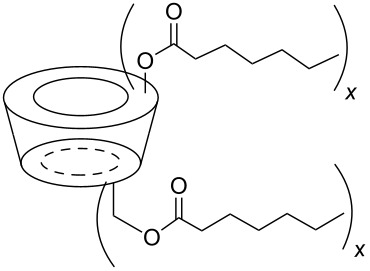		

Subs.	x = 3–8	x = 4–7	x = 14–21	x = 2–4	x = 5–11	x = 10–14
DS	5.0	6.7	18.1	3.5	8.4	12.1
*M*_w_	1629.77	1798.21	2927.74	1481.15	1966.65	2333.35

Six VOCs (Table 2) were added to a 20 mL glass vial containing 10 mL of water and a piece of coated PP. Their presence in gas phase was measured by static headspace gas chromatography after a minimum equilibration time of 12 hours. After this, equilibrium between the three phases of the VOCs, gaseous, aqueous, and complexed, is assumed to be established according to:

[1]
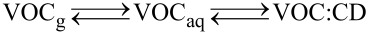


where VOC_g_ and VOC_aq_ are the VOCs present in the gaseous and aqueous phase of the GC vial, respectively, and VOC:CD is the adsorbed concentration of the VOCs by the ACD functionalised PP. When analysing the samples with SH-GC, the resulting area under the curve (AUC) will be proportional to [VOC_g_], which in turn is proportional to [VOC_aq_]. A standard calibration curve was constructed from known concentrations of VOCs added to vials containing the same amount of water as in the experiments, which allowed the distribution ratio between gaseous and aqueous phases to be found. Hence, the concentration of VOCs adsorbed by the coated PP can be deducted directly from the AUC by:

[2]
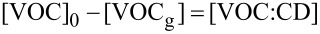


where VOC_0_ is the initial amount of VOC added to each vial, VOC_g_ is the amount of VOC detected by GC in the gaseous phase, and VOC:CD is the amount of VOC adsorbed onto the PP sheet.

It was previously shown that the coating obtained by the dip method described above is stable in the presence of water and in fact, it renders the coated material more hydrophilic [21]. Polypropylene is a very hydrophobic polymer and coating with ACDs makes the textile wettable, which in turn makes it a suitable support material for the coating even in water purification experiments as described here. In other words, coated PP pieces were fully submerged in the water phase for the entire experiment as opposed to non-coated PP pieces which were not wettable and, therefore, only partly submerged. This made the comparison between the adsorption of the VOCs by coated and non-coated PP problematic, as the former was an expression of aqueous adsorption, while the latter was of gaseous adsorption. Furthermore, it has been shown that the ACD coating is equally distributed along the PP fibres [22]. Hence, it could be argued that no crude PP fibres are present in a coated PP sample and, therefore, no adsorption by the untreated PP itself. For these reasons, it was decided to view the adsorption of the coated PP samples as a combination of the adsorption caused by the coating together with the potential, possible adsorption of crude PP fibres (if present), and accordingly no direct comparisons were made.

When preparing the coating solutions using the various ACD derivatives, clear differences in the particle sizes in the solutions/suspensions could be seen. The ethanol concentration required to keep the ACD derivatives in solution is dependent upon the particular derivative in question (Table 4). This can be explained when looking at the DS of the ACD derivatives. As expected, **9** (DS: 18.1) requires more ethanol to stay in solution than **10a** (DS: 3.5) since **9** is more hydrophobic than **10a**. However, **7** (DS 5.0) and **8** (6.7) are very similar and yet required the opposite content of ethanol to obtain the highest uptake.

**Table 4:** Phase distribution in the coating solution at various ethanol/water ratios. “–“ : clear solution; “x” : opaque suspension.

Ratio ethanol/water	**7**	**8**	**9**	**10a**	**10b**	**10c**

100	–	–	–	–	–	–
80/20	–	x	x	–	–	–
60/40	–	x	x	–	x	x
40/60	x	x	x	x	x	x
20/80	x	x	x	x	x	x

**Figure 1:**
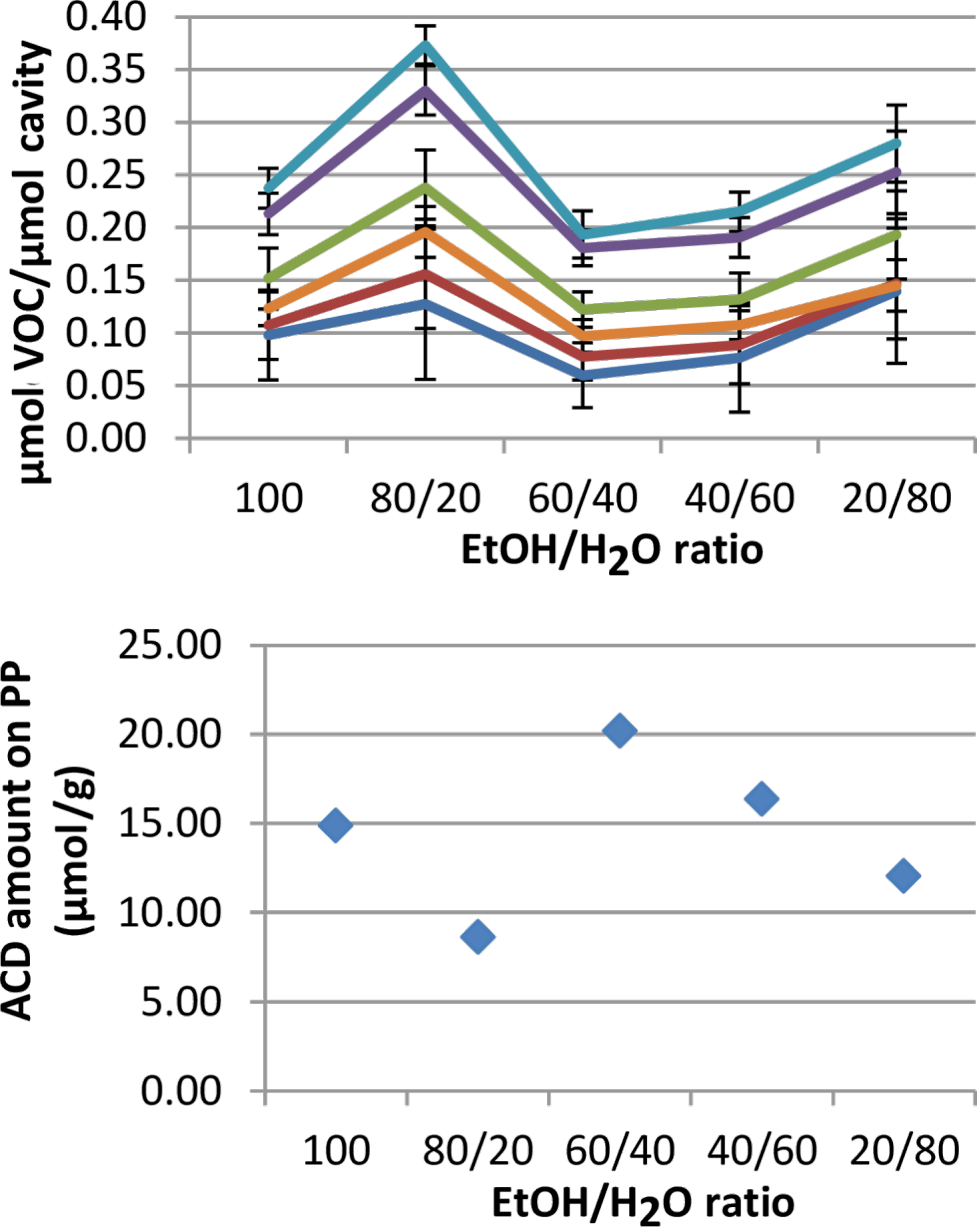
Uptake selectivity (left) compared to weight gain of coated polypropylene (right) exemplified by a coating of ACD **2**. Standard deviation (*n* = 3) indicated by the error bars.

It is possible to control the amount of the coating on the PP by changing the concentration of the ACD derivative and/or the ethanol/water ratio. Previous studies proved the coating amount of PP to be fairly reproducible [21] and as the present study is focused on the uptake capacity, the results were calculated as µmol VOC per µmol CD cavity present on the PP sheet in question, that is, as a VOC:ACD ratio. Looking solely at the amount of coating achievable, the optimal ethanol/water ratio was shown to depend on the support material of choice: for PP the optimal ratio was between 40/60 and 60/40 [21]. However, if for some reason the cavities of the ACDs are inaccessible for inclusion complex formation, it would not be cost-efficient to simply focus on obtaining the maximum coating amount. For this reason, the uptake capacity of the coating obtained from a coating solutions of ethanol/water = 100, 80/20, 60/40, 40/60 or 20/80 was investigated. One ppm of each VOC was added to the GC vials containing the coated PP, corresponding to a total amount of added VOC of 0.444 µmol/vial. Figure 1 uses **8** (per-O6-substituted, DS 6.7) to illustrate the results of the experiment. The graph on the right shows the amount of ACD present in the vials and is thereby an indication of the effect of the ethanol/water ratio on the coating amount. These results were used to calculate the VOC:ACD ratio as represented in the graph on the left. As the purpose of the study was to observe the uptake capacity of ACD-coated PP in a complex solution of pollutants, it was therefore more interesting to look at the combined uptake of all the VOCs. The VOC:ACD ratios obtained from the experiments were summarised and the results from each ACD derivative were compared with one another as illustrated in Table 5. A tendency that certain ethanol/water ratios resulted in a greater uptake of VOCs was observed. For instance, **8** has the highest adsorption of VOCs per ACD (µmol/µmol) when the coating is assembled from a coating solution with an ethanol/water ratio of 80/20, while **7** requires an ethanol/water solution ratio of 20/80. The comparison between Table 4 and Table 5 shows that the highest uptake of the VOCs is obtained when the coating is assembled from suspensions of ACDs rather than solutions. It was previously hypothesised that the ACD derivatives order themselves in channel structures on the surface of the support material [21]. It is likely that these channel structures were the reason for the observed preference for certain ethanol/water ratios. That is, if the environment in which the coating assembly takes place is less than optimal, the coating would not assemble as an optimized structure, which would then diminish the uptake capacity as illustrated in Table 5. When looking at Table 4 and Table 5, it seems that the randomly substituted ACD derivatives have similar properties independent of their individual degree of substitution. They are all fairly soluble in water and maximum capacity is achieved when using a coating solution consisting mainly of water. As previously discussed [21], it is hypothesised that the location of the substituents influences the formation and possibly the constitution of the channels. In this context, it can similarly be hypothesised that the common features of the randomly substituted ACD derivatives are linked to a similar structure of the channels caused by the fact that the substituents are located on both rims of the CD. As mentioned above, the maximum coating amount obtained on PP resulted from coating with a solution of fairly equal amounts of ethanol and water. However, the present results indicated that quantity is no guarantee for quality when it comes to securing the highest uptake capacity.

**Table 5:** Summarised VOC/cavity ratio (µmol/µmol) calculated for the uptake of the six VOCs on polypropylene sheets coated with the various six amphiphilic CD derivatives using five different ethanol/water ratios. The maximum values are highlighted in bold.

Ratio ethanol/water	**7**	**8**	**9**	**10a**	**10b**	**10c**

100	0.83	0.93	1.57	1.12	1.36	1.61
80/20	1.26	**1.42**	**2.67**	1.32	1.30	1.21
60/40	0.68	0.73	0.49	1.40	1.14	0.99
40/60	1.82	0.81	1.00	**2.23**	**1.97**	0.98
20/80	**2.39**	1.16	2.40	1.23	1.43	**2.59**

All of the ACD derivatives showed the same tendency towards uptake selectivity of the VOCs: **5**, **4**, **3**, **6**, **2**, and **1**, ordered from highest to lowest cavity affinity, see Figure 1. It is known that one of the driving forces in inclusion complex formation in water between CDs (the host) and the included compound (the guest) is the hydrophobicity of the guest [7]. Hence, there is an inherent tendency towards the formation of inclusion complexes with **5** in comparison with **1** (see Table 2 for reference to hydrophobicity of the VOCs, represented by the log *P* value). Although butylbenzene (**6**) was expected to have the highest affinity towards the cavity due its hydrophobicity, it seems not to be as prone to complex formation as anticipated.

As briefly mentioned above, the uptake capacity relies on the ACD derivative used and likely also on the correct assembly of the coating. When looking at the most favourable coat with respect to the maximum uptake of the VOCs, an investigation of a potential selectivity of the ACD derivative is possible (see Table 6). The uptake of each of the VOCs by the different ACD derivatives is stated in µmol VOC/µmol CD cavity. The derivatives are ordered from lowest DS (**10a**) to highest DS (**9**). There does not seem to be any difference in the selectivity of the various ACD derivatives. If the alkyl side chains of the CD would have played a role in the formation of the inclusion complexes between the CD cavity and the VOCs, for instance by elongating the cavity, it would be reasonable to expect some difference in the selectivity especially between **10a** and **9** due to their different DS of 3.5 and 18.1, respectively. There is, however, a small difference in the capacity when comparing **9** and **10c** with **10a** and **7**, in other words, when comparing highly substituted ACDs with low-substituted ACDs. The fact that **9** and **10c** have a higher uptake capacity but the same selectivity as the other derivatives could indicate that the alkyl side chains play a part in the combined uptake capacity of the coating, perhaps due to the alkyl chains forming an apolar phase between the cavities. It could then be speculated that the higher DS, the greater the number of chains, thus a more dense coating, resulting in a higher uptake capacity, which would then explain the differences in the uptake capacity between **9** and **10c** (2.7 µmol VOC per µmol ACD) and **7** and **10a** (2.4 and 2.2 µmol VOC per µmol ACD, respectively), as seen in Table 6. In other words, the combination of the hydrophobic alkyl chains and the CD cavity seems to provide a coating with a very high capacity for uptake of VOCs.

**Table 6:** Maximum uptake (µmol VOC/µmol cavity) of the various VOCs by each amphiphilic CD derivative. The amphiphilic CD derivatives are ordered by ascending DS.

	**10a**	**7**	**8**	**10b**	**10c**	**9**

**1**	0.364	0.219	0.127	0.323	0.460	0.460
**2**	0.341	0.263	0.156	0.312	0.395	0.407
**3**	0.384	0.399	0.238	0.383	0.438	0.450
**4**	0.432	0.549	0.330	0.484	0.561	0.511
**5**	0.435	0.619	0.373	0.505	0.582	0.516
**6**	0.261	0.331	0.196	0.281	0.264	0.314

SUM	2.217	2.380	1.420	2.289	2.700	2.658

**Figure 2:**
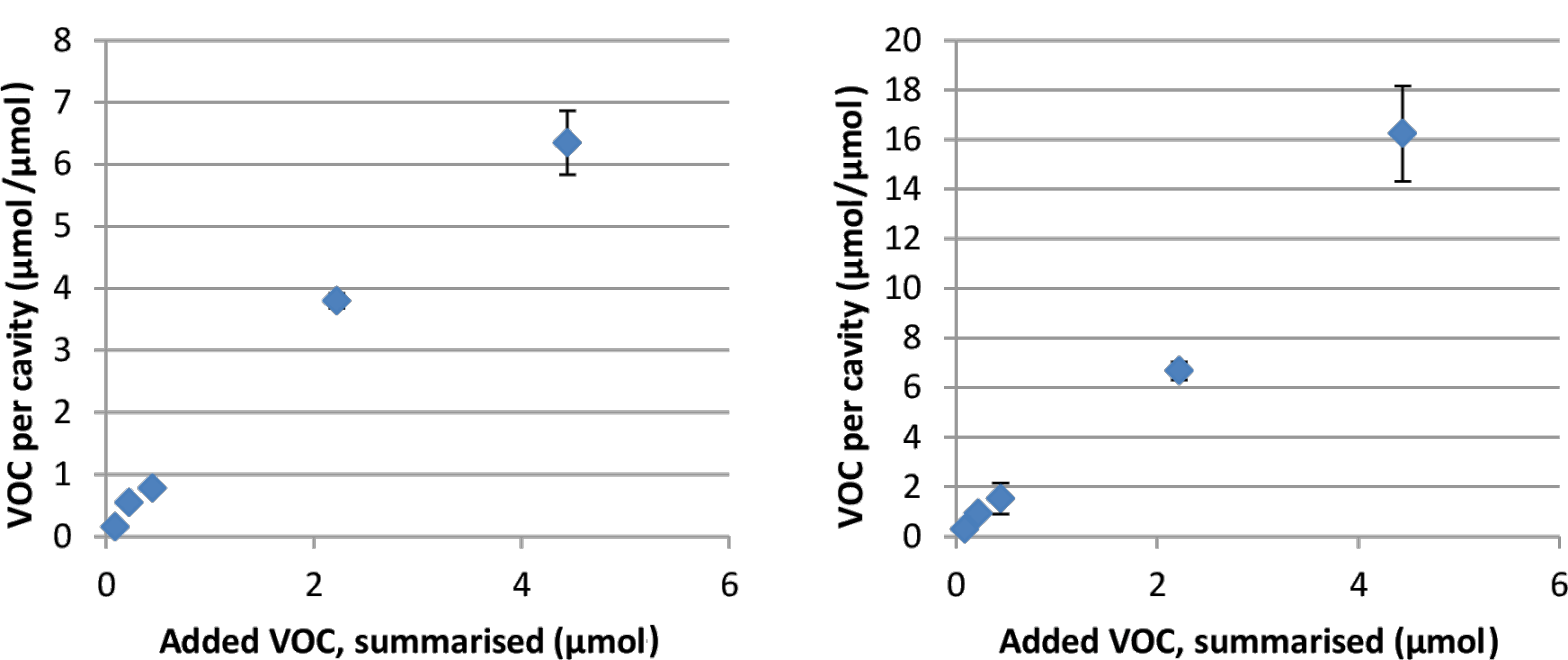
Results of titration experiment for **8** (left) and **10c** (right). Standard deviation (*n* = 3) indicated with error bars.

This hypothesis was further supported by a titration experiment. VOCs were added to the vials ranging from 0.2 ppm to 10 ppm, corresponding to approximately 80% of the CD cavities present in the vial to approximately 20 times as many VOC molecules as CD cavities per vial. Usually, when working with CDs in aqueous solutions, inclusion complexes of relatively small stoichiometries, such as 1:1, 1:2, 2:1, etc., are expected [8]. Although the derivatives are ordered in multilayers [22], it was anticipated that the adsorption would somewhat follow the Langmuir isotherm. This seemed logical as each CD monolayer was assumed to be independent of the other without interaction between the adjacent adsorbed VOCs and more importantly, a saturation point must exist at which point the coating cannot further adsorb VOCs. Hence, it was predicted that a surplus of 20 times the amount of VOCs to the amount of cavities would be more than enough to reach the saturation point of a typical Langmuir isotherm. However, this was not the case. None of the six ACD derivatives showed signs of reaching saturation within the conditions of the experiments, indicated by a linear isotherm plot. An interesting part of the experiment was the indication of the uptake capacity of the coating. As mentioned above, a stoichiometry ratio of around 1:1 is expected in terms of CD inclusion complexes. However, Table 3 actually indicates that the ACD coating is capable of removing more VOCs than typically expected and the titration experiment also showed that the coating has an uptake capacity corresponding to guest–host stoichiometries from 6:1 (**8**) up to 16:1 (**10c**) (see Figure 2). Since the saturation point was not reached during these experiments, the results indicate that further addition of VOCs would result in even higher stoichiometries. However, a stoichiometry of even 6:1 for β-CD:benzene/benzene derivatives is, of course, not caused by inclusion complex formation alone, as that would be physically impossible. The large uptake capacity of the coated PP must, therefore, be caused by phenomena other than inclusion complexes alone.

The observation of the large capacity for VOC reduction led to an experiment in which the uptake of the ACD coating was compared to the uptake of the equivalent amount of native β-CD in solution. One ppm of VOC was added to each vial and the total amount of VOCs adsorbed per cavity (µmol/µmol) was calculated. The results were stated as an ACD-functionalised PP versus β-CD ratio, as shown in Table 7. As can be observed, immobilising β-CD on PP has a remarkable effect on the capacity: between 16 and 104 (mean = 50, σ = 26) times more VOCs are removed from the aqueous phase if the β-CD is immobilised on PP rather than β-CD in solution. This is, of course, probably due to the added dimension consisting of the addition of the alkyl chains. Furthermore, **6** was only adsorbed by the ACD coating, and not by the native β-CD. This further supports the hypothesis that the CD cavities and the alkyl side chains combine to form a very potent coating for the uptake of VOCs from an aqueous phase.

**Table 7:** Comparison of the uptake of VOCs for the amphiphilic coating and native β-CD in solution. The numbers are the ratio between the uptake of the ACD coating and the uptake of β-CD. Native β-CD did not remove any **6** from the gaseous phase.

	**7**	**8**	**9**	**10a**	**10b**	**10c**

**1**	29	16	57	45	40	22
**2**	33	23	61	51	47	30
**3**	52	51	96	82	82	59
**4**	58	67	104	88	98	73
**5**	18	22	31	26	30	22
**6**	–	–	–	–	–	–

As mentioned above, we previously hypothesised that the functionalisation method presented in this study results in the formation of multiple, alternating layers of CD cavities and apolar alkyl chains. The present results support this theory as the uptake capacity of the coating exceeds the expected capacity of both native CDs in solution and other functionalisation methods in the literature (see Table 1 for literature references). The ability of the amphiphilic CD coating presented here is not dependent on the choice of CD, which means that α- and γ-CDs can be used equally well. A combination of α-, β-, and γ-CDs would most likely enhance the selectivity of the coating as the inclusion complex formation is, amongst other things, dependent on the cavity size. The potential use in water filtration applications is significant as this coating technology is simple and readily implementable in existing production lines, as previously reported [21]. Furthermore, to our knowledge no other method for functionalising surfaces with CDs results in an uptake capacity of the magnitude reported in this study.

## Conclusion

These results show that the tested amphiphilic CD derivatives are quite capable of removing small pollutants from water. As seen in the literature, CDs are able to include a vast number of compounds of varying size in the cavity, which makes them very suitable for commercial application in water purification. With an average uptake capacity of 50 times that of native β-CD in solution, the technology presented here provides a more potent alternative (as compared to other technologies previously presented in the literature) for industrial application for water purification.

## Experimental

### Materials

Untreated, isotactic polypropylene was kindly provided by Fibertex A/S (Aalborg, Denmark). Benzene and benzene derivatives (Table 2) were purchased from Sigma-Aldrich (St. Louis, Mo, USA). ACD derivatives were synthesised as described previously [8]. Purified water was obtained as de-ionised water.

### Preparation of amphiphilic cyclodextrin-coated polypropylene

Pieces of PP were cut into suitable sizes, washed in warm ethanol and dried prior to use. Coating solutions consisting of 1 mg/mL of amphiphilic CD derivative and ethanol/water ratios of either 100/0, 80/20, 60/40, 40/60 or 20/80 were prepared by dissolving the CDs in ethanol prior to the addition of water, if required. The PP pieces were dipped for approximately 10 seconds into the coating solution, using 30 mL solution per gram PP, and left horizontally to dry overnight on a steel mesh at room temperature. All experiments were performed in triplicate. The weight was noted before and after the coating procedure and the quantity of amphiphilic CD on the PP was determined by:

[3]
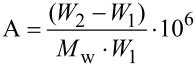


where *W*_1_ and *W*_2_ are the weights (g) of the PP prior to and after the coating procedure, respectively, *M*_w_ is the average molecular weight (g/mol) of the amphiphilic CD, and A is the amount of the coating as expressed in µmol/g.

### Static headspace gas chromatography analyses

The VOCs (Table 2) were prepared as 1000 ppm ethanol solutions. 10 mL of water containing the suitable amount of VOC solution and a 12.5 cm^2^ piece of PP were introduced into 20 mL glass vials and sealed with a silicone septum and aluminium foil and left overnight. Measurements were conducted with an Agilent G1888 headspace autosampler. A 1 mL sample was withdrawn from the vial using a gas-tight syringe and injected directly into the chromatography column via a transfer line (250 °C) and analysed by gas chromatography (Perkin Elmer Autosystem XL) equipped with a flame ionization detector using a DB624 column. The detector temperature was 280 °C, the column temperature was 120 °C and the run time was 8 minutes.
